# General Health as Expressed by the Work of the Kidneys, and Its Relation to the Teeth

**Published:** 1887-11

**Authors:** Chas. Mayr

**Affiliations:** Springfield, Mass.


### GENERAL HEALTH AS EXPRESSED BY THE WORK OF THE KIDNEYS, AND ITS RELATION TO THE TEETH.

Read before the Connecticut Valley Dental Society at Montreal, P. Q., July 22d, 1887.

BY PROF. CHAS. MAYR, SPRINGFIELD, MASS.

While connected with the *New England Journal of Dentistry* I had ample opportunity to observe the importance which was attached by all essayists on general dental pathology to the relation between general health and strength of teeth, meaning by the word strength, not only mechanical hardness, but the general power of resistance. As I have said in former essays, the life of the tooth, as the life of the individual, is the resultant, to use a physical term, between the forces within the tooth and the individual, and the forces outside of the tooth and the individual. The many various agents which tend to destroy teeth are similar to the causes which destroy individuals; either excess of the strength of the outer attack or deficiency in the means of defense, and finally what might be called its "kismet." The tooth is influenced by everything going on in the organism to which it belongs, in a degree which we hardly yet realize.

It is now so long since the living structure of the tooth was demonstrated, that I need scarcely tell you not to think a tooth a senseless block of marble. Some of you have read that fine essay of Flammarion, who imagined an astronomer with a very big telescope stationed on one of the stars, so far distant that just now the light of deeds and happenings on our globe thousands of years ago is just reaching him. He sees everything as plainly as if he stood but a mile or two away, because his telescope magnifies quintillions of times. In the same manner we may say that if we could have microscopes, chemical tests and acuteness of perception quintillions of times better than we now have, we would see in every tooth the history of its development through our Simian ancestors, the Marsupials, the Saurians, etc., down, pointed out as closely as if they were written in black and white. It is only the small brain of a small people, with but small thoughts who know all about this world, but the real, actual world of ours is so deep that the greatest intellect is nothing but a yard stick with which we would sound the depth of oceans. But by industry and intelligence we may add a little to our yard stick every century, and thus gradually sound deeper and deeper. We can as yet only try around the shores, but our chartography will become more complete with added experience and knowledge. Those enquiries which enlarge our conception of the depth and infinity of things cannot help but elevate us, however little of dollars and cents we may see in them at present. Having thus, in a general way, indicated the general line of thought along which I try to work out problems, and from which I derive some satisfaction, I shall try to increase our knowledge of the points of relation existing between teeth and general health.

The food which we take into our bodies is more complex than that which we secrete; sugar, bread, starch, part of the woody fiber, the different kinds of vegetables, disappear without pronounced specific compounds. All kinds of meat and albuminoid food yield but a small number of compounds, and happily in a form which allows the chemist to examine them more carefully than almost any other waste product of man. From time immemorial the excretions of the human system have been considered offensive and vile, but looking at the matter from a purely chemical standpoint, they are much less so than many chemicals we have to deal with. The secretion of the kidneys—urine especially—is a fairly clean solution of a number of distinct chemical compounds. To understand somewhat their composition, I shall review very briefly the chemical compounds that are characteristic and have been discovered in urine.

The average daily excretion of an individual varies from one pint to two quarts and more, but the amount of solid chemical compounds produced in the same time is far more constant, varying from one and one-half to two ounces in the adult human being. As a rule, many physicians have so far forgotten their knowledge of urinary analysis that they can only take what is called the specific gravity, a point of information of but very little value. But in spite of all the different products, I myself found and verified the fact that the total of solids bears a remarkably close relation to specific gravity.

If, for instance, we find urine of a specific gravity of 1020, we will get a very close approximation of the percentage of solids dissolved by multiplying the last two figures by 0.23, giving us in this case 4.6 per cent. This rule does not hold good in pathological urine. A specimen of a specific gravity of 1040, for instance, ought to give, according to this rule, 9.2 per cent. solids, while it was found to contain only 8.4 per cent., being a diabetic urine. Another case of specific gravity 1006 ought to have contained 1.38 per cent. solids, while it contained 2.60 per cent, it being a case of Bright's Disease. Of the solid substances occurring most frequently in urine, I will mention only urea, uric acid, creatine, and the mineral salts of the food as having common salt sulphates, phosphates, etc.

Text-book makers say that the normal reaction of urine is acid. I fail to see the truth of this statement. If we test with an imperfect reagent like litmus, perhaps we will have a faint reddening due to carbonic acid, but if we use more reliable tests like carmine, and some other agents, we will find that the reaction is acid only as far as the carbonic acid gas prevails, but decidedly alkaline in regard to solid constituents.

The statement that fresh urine reacts acid is, like many book statements, but partly true. An alkalicity of one-tenth of one per cent. is not rare. Of the chemicals found in urine, the most interesting, and the one to which I shall confine myself, is urea, a substance which was the first organic compound made from entirely inorganic substances outside the body. Urea, pure, tastes like saltpetre. Its composition is $C\,O\,N_2H_4$.

As a rule, urine contains from one to four per cent. of this salt. When urine become putrescent, this compound decomposes into carbonic acid and ammonia. Uric acid is a fine, clear powder, of very odd shapes when viewed with a microscope. Creatinine is derived from the creatine in meat, and seems to be excreted without any other change. As a rule, lime-salts, lactic acid, oxalic acid, etc., are found in urine. The urea excreted is a measure of the person's activity, far more reliable than any other indication. About two hours after some intense activity, a maximum of excretion takes place, which sometimes goes as high as 2900 milligrammes per hour after very heavy work, or at the rate of 64 grammes daily. On the other hand, during the morning hours, the average falls as low as 500 milligrammes, or at the rate of only 12 grammes a day. It is not alone bodily activity which influences this waste, for mental activity has the same effect. I have a series of very interesting observations which show how the fluctuations compare with the work, and it is astonishing to observe how thought expresses itself in urine.

Between the different chemicals excreted there exists a certain very striking relation, which may be said to be the health relation of the excretions. If we call the quantity of urea excreted 100, that of phosphoric acid will be about 8., of uric acid 5.6, of lime 2.1, and of salt an amount varying from 58 to 63. Any pronounced disturbance of these relative amounts is accompanied by a pronounced disturbance of the general nutrition of the body. A high percentage of urea indicates too much waste work in the body, because it is not only the visible work which appears as urea, but the worrying, restless work by which many otherwise hopelessly lazy people are tormented. A high percentage of uric acid is always accompanied with dyspeptic symptoms.

At the meeting of the American Dental Association in Saratoga a few years ago, some one said that in all cases of pyorrhœa alveolaris which had come under his observation, he had found a uric acid, or gouty diathesis. Perhaps a urinary analysis would have cleared up some points in a case which has come under my own observation. A remarkable absorption of the teeth was observed, and the urine was found to contain large quantities of lactic acid and lactate of lime. In such instances there is very little hope from mere local treatment or from filling the teeth, but a constitutional treatment might rapidly produce a complete change. We all know how constantly extensive caries accompanies diabetes, a disease most readily diagnosed by examination of the urine, and how teeth seem to lose lime-salts during pregnancy. Do these lime-salts go to the fœtus, or are they excreted? An analysis of urine may solve the problem. Is pyorrhœa alveolaris always connected with excess of uric acid? How does the oxalic acid formation in the system and its excretion affect teeth? These are a few of the questions which will present themselves to the thoughtful practitioner in connection with the subject, and which can only be approached by his coöperation with those able carefully to investigate the matter. The main object in the writing of this paper is to obtain the coöperation of dental practitioners in the obtaining of specimens of urine for analysis, in which there are peculiar constitutional complications accompanying great disturbances of the teeth.

